# Higher socioeconomic deprivation is associated with regional vulnerability to atrophy in hippocampal subfields and amygdala in a real‐world population with mild cognitive impairment and dementia

**DOI:** 10.1002/alz70856_100670

**Published:** 2025-12-25

**Authors:** Natasha V Stevanovich, Robert Stewart, Dag Aarsland, Gayan Perera, Ashwin V Venkataraman

**Affiliations:** ^1^ Centre for Healthy Brain Ageing, IoPPN, King's College London, London, United Kingdom; ^2^ South London and Maudsley NHS Foundation Trust, London, United Kingdom; ^3^ Centre for Healthy Brain Ageing, IoPPN, King's College London, London, London, United Kingdom

## Abstract

**Background:**

Socioeconomic deprivation is associated with increased risk of co‐morbid diseases that are modifiable risk factors for dementia, and reductions in hippocampal and amygdala volume, key regions in neurodegenerative pathology. To date, these changes have not been studied in real world populations of cognitive impairment. Machine learning approaches now enable visualisation of smaller structures such as hippocampal subfields from routine clinical MRIs, previously not possible. This study investigates the association between socioeconomic deprivation, and *apriori* selected volumes (whole brain, hippocampus, hippocampal subfields, amygdala) in patients with cognitive impairment.

**Method:**

Patients from South London and Maudsley (SLaM) Image Bank diagnosed with MCI/dementia subtypes with available MRI scans were identified. UK Index of Multiple Deprivation (IMD) scores, modifiable dementia risk factors (hypertension, diabetes, cerebrovascular accident and asthma) were obtained. Multiple linear regression models adjusted for age, gender, ethnicity, and modifiable risk factors were compared with hippocampal subfields, whole hippocampus and amygdala (ANTsX pipeline ‐ Deepflash with the DSK atlas applied, corrected for total intracranial volume) with stratified analyses for cognitive impairment subtypes.

**Result:**

3,075 patients (mean age 77, 58% female, 40% minoritized ethnicity, AD (*n* = 2003), MCI (*n* = 660), VaD (*n* = 239), other/unspecified (*n* = 173) were included. Higher IMD (greater deprivation) correlated with significantly reduced hippocampal and amygdala volumes across all cognitive impairment types. For every 10‐point increase in IMD, hippocampal volume decreased by 76 ml (left) (*p* = 0.002) and 104 ml (right) (*p* <0.001), CA1 subfield volume by 46 ml (left) (*p* <0.001) and 43 ml (right) (*p* = 0.001), parahippocampal gyrus volume by 58 ml (left) (*p* = 0.011) and 51 ml (right) (*p* = 0.034), perirhinal cortex volume by 37 ml (left) (*p* = 0.046) and 44 ml (right) (*p* =  0.001), and amygdala volume by 12 ml (left) (*p* = 0.004) and 14 ml (right) (*p* <0.001). The strongest associations were observed in AD and MCI groups.

**Conclusion:**

We show higher deprivation is linked to distinct hippocampal subfield and amygdala atrophy in cognitive impairment. Socioeconomic deprivation may have a larger role in reflecting regional vulnerability to neurodegeneration than previously thought.

